# Gender disparities in the education gradient in self-reported health across birth cohorts in China

**DOI:** 10.1186/s12889-020-08520-z

**Published:** 2020-03-20

**Authors:** Bowen Zhu, Yiwan Ye

**Affiliations:** 1 School of Public Administration, Hunan Normal University, Lushan Road 36, Changsha, 410081 Hunan China; 2 Department of Sociology, University of California, Davis, 286 Social Science & Humanities Building, Davis, 95616 USA

**Keywords:** Education and health gradient, Gender disparity, Cohort effect, Latent growth-curve model

## Abstract

**Background:**

Variation in the relationship between education and health has been studied intensely over the past few decades. Although there is research on gender disparity and cohort variations in educational effect on health using samples from the U.S. and Europe, research about China’s is limited. Given the specific social changes in China, our study is designed to analyze the gender and cohort patterns in the education-health gradient.

**Method:**

The latent growth-curve modeling was used to analyze the gender and cohort variations in the education gradient in self-rated health among Chinese respondents. The study employed longitudinal and nationally representative data from the Chinese Family Panel Studies from the years 2010 to 2016. Each cohort is specified according to their distinct periods of social change in China. Following the analysis, we used latent growth-curve model to illustrate gender and cohort differences in the age-graded education and health trajectories.

**Results:**

Although Chinese men have reported to have better health than women in general, women reported 1.6 percentage points higher in self-reported health for each additional year of schooling compared to that of men (*P* < 0.001). The latent growth curve model showed women’s extra education benefits were persistent overtime. Compared to the people born during the “Old China” (1908–1938), the education gradient in self-rated health did not change for cohorts born before 1955 and after 1977, but the education-health gap changed significantly in the 1956–1960 (O.R. = 1.038, *P* < 0.05), 1967–1976 (O.R. = 1.058, *P* < 0.001), and 1977–1983 (O.R. = 1.063, P < 0.001) cohorts. There was a gender difference for the cohort variations in the education-health gradient. For women, the education effect in the 1956–1960 (O.R. = 1.063, *P* < 0.05), 1967–1976 (O.R. = 1.088, *P* < 0.001) and 1977–1983 (O.R. = 1.102, *P* < 0.001) cohorts was significantly higher than that of the 1908–1938 cohort. On the contrary, the education-health gradient remained the same across all cohorts for men.

**Conclusion:**

Our study suggests that the education-health gradient varies across cohorts for women, but the size of education effect remains consistent for men across cohorts. The findings support the resource-substitution hypothesis and not the rising-importance hypothesis in China. We discussed the potential influences of the unique, social transformation and educational expansion in China.

## Background

Over the past few decades, the relationship between education and health or education-health gradient has been studied intensely. Education was found to affect health directly and indirectly through increasing income and improving job outcomes [1, 2], enabling a healthy lifestyle and strong social support [3], supporting a sense of control in life, enhancing confidence in problem-solving, and strengthening the ability to cope with stress [4, 5]. Although education’s positive effects on health are well-studied, it is also important to analyze which population subgroup benefits more from it and which group does not as well as if there are any changes in the education-health relationships across birth cohorts.

The gender gap in health attracts considerable attention from researchers [6–12]. Their studies show that men tend to report better health experiences and outcomes than women despite higher life expectancy for women versus men. Social factors – underlying social advantage or disadvantage - rather than biological factors are identified as primary explanations for the gender difference in health [13, 14]. Despite advances in gender equality over time, women remain socially and economically disadvantaged in comparison with men, and there are still substantial limits in access to health-related resources for women [15, 16]. Compared with men, women face restricted opportunities for paid employment, higher wages, fulfilling work, and authority in the workplace [10]. According to the ‘resource substitution hypothesis’ proposed by Ross and Mirowsky [9, 10], resource substitution exists when having multiple resources make outcomes less dependent on the presence of any specific resources and it implies that education’s influence on health is greater for persons with fewer alternative resources than it is for the more advantaged. Women’s disadvantaged status means that they generally have fewer resources than men. According to the resource substitution hypothesis, women depend more heavily on education to improve their health. The present paper examines whether the resource substitution hypothesis is supported and whose health benefits more from education in the Chinese context.

Social transformation also shapes the relationship between education and health. Throughout the twentieth century, significant social changes have occurred in many countries around the world, especially in developing countries like China. Due to dramatic social changes that shape cohort differences, both the ‘rising importance hypothesis’ [17], and the ‘diminishing health returns hypothesis’ [18–20] have been tested. The rising importance hypothesis suggests the positive education effect on health increases across birth cohorts, whereas the diminishing health returns hypothesis suggests the education effect diminishes across birth cohorts. Subsequent findings on gender difference and cohort variation reveals both gender and cohort-specific social context shapes education gradient in health. However, these findings are based on the Western democratic context – using data from the U.S. and Europe [17–22]. Empirical work on gender and cohort effect and the education-health gradient are often absent in the Chinese context, especially after the educational expansion therein. Our study is to expand on the knowledge if similar gender and cohort patterns of education-health gradient occur in the Eastern communist state.

Compared to the U.S. and Europe, the association between education and health-related resources was more complicated in China due to its drastic socio-political transformation – such as the civil war (1946–1950), the Great Famine (1959–1961), the Cultural Revolution (1966–1976), and Economic Reform (1979–1989). In U.S., the positive relationship between education and income had intensified over the years [23], and growing income difference due to education further increased the health gaps in recent cohorts [21]. In Europe, from 1960s onward, participation in higher education had increased, however, the massive growth in tertiary education has not been accompanied by an equivalent growth in the labor market [18, 19]. Unlike their Western capitalist counterparts, China prior to the marketization reform in 1979 was a collective economy where the government assigned salaries and occupations to individuals, making educational attainment irrelevant for job acquisition [24]. During the Reform and Opening-Up periods, it was not uncommon for people without higher education to earn high wages, thereby explaining the weakness of the education-income relationship. However, from 1992 to 2004, the wage returns to education rose steadily and then stagnated, and ultimately declined from 2004 to 2009 due to the educational expansion [25].

Not only was the gendered relationship between education and health-related resources in China idiosyncratic, but also the cohort variations in education. From 1966 to 1976, the Cultural Revolution had a devastating influence on education, especially for higher education as demonstrated by the college entrance examination system shutting down during that period [26]. According to the Chinese Educational Statistical Report, there were only 0.85 million college graduates in China in 1999, but four years later, the number of graduates rose to 1.88 million. By 2017, there were around 7.36 million college graduates in China [27]. The likelihood of female high school students getting into colleges had been reported to be the similar to that of male high school students [28]. However, the significant educational advancement in China resulted in a market devaluation of educational credentials, and the influx of college credentials also made the labor market more competitive [29]. Gender gap in education was also influenced. It was not until the policy implementation of higher education expansion in 1999 that increased the opportunity of receiving a college education and narrowed the gender gap in educational attainment [30]. However, the gender gap in receiving formal education, junior high school persists [28]. Additionally, urban female residents received more benefits from the education expansion, and gender inequality in education even increased in rural areas [31]. These social upheavals and policy changes in China occurred at different time points, and therefore their influences may vary for birth cohorts who came of age in different historical periods. This study aimed to examine the education-health gradient from both gender and cohort perspectives in the Chinese context.

Data from the Chinese Family Panel Studies (CFPS) are used to answer the following research questions: (1) Is the education benefit in health larger for men or women? I.e. will ‘resource substitution hypothesis’ be proved in China? (2) Is there any inter-cohort variation in the association between education and health? I.e. will ‘rising importance hypothesis’ or ‘diminishing health return hypothesis be supported in China? (3) Is there any gender difference in inter-cohort variations in education-health gradient?

## Methods

Our analytic strategy includes two steps. Firstly, we present the descriptive statistics of our analytic samples to illustrate the characteristics of our main variables of interests, namely self-rated health, education years, gender, and other keys covariates. Secondly, we used the latent growth rate model to test the beforementioned hypotheses. This model allows us to examine the statistical significance of education effect on health across gender and cohort groups while controlling for covariates, within individual change, and other unaccounted random errors.

### Sample

We used four waves of Chinese Family Panel Studies data (2010, 2012, 2014, and 2016) from a nationally representative survey of adults aged 18-years and older. The surveys were administered by the Institute of Social Science Survey at the Peking University of China and were designed to study the historical change of society, economy, population, education, and health in China. The survey used computer assisted personal interview to collect panel data at the individual, household, and community levels. Respondent selection was guided by implicit stratification and multi-stage probability proportional to size (PPS) sampling. The unit in the first sampling stage was a county, the unit in the second stage was a neighborhood committee, and the unit in the final stage was a family household. In the first two sampling stages, the official administrative division data were used to select the counties and neighborhoods, and the households were sampled using the cyclic isometric method with random starting points. The panel design provides an opportunity for cohort analysis of social and economic change over time. We identified the sample of adults aged 22 years or older in 2010 as the baseline cohort, and the working sample consists of 23,706 individuals in 2010, 26,094 individuals in 2012, 25,724 individuals in 2014, and 25,084 individuals in 2016. In total, there were 27,580 unique respondents and 100,608 observations between 2010 and 2016. The percentage of individuals surveyed four to six times is 65.89%, and the percentage of individuals surveyed only three times is 34.11%.

### Variables

The self-reported health variable measures respondents’ subjective assessment of their health. Respondents were specifically asked, ‘How good is your health in general?’ The Likert scale in 2010 includes the response options of ‘very bad,’ ‘bad,’ ‘a little bad,’ ‘fair,’ and ‘good.’ We coded the first of these four items as ‘0’ and “good” health as ‘1.’ The Likert scale from 2012 to 2016 includes the response options of ‘bad,’ ‘fair,’ ‘little good,’ ‘good,’ and ‘very good.’ The first two items are coded as ‘0,’ and the last three answers are coded as ‘1.’ The self-reported health measure is regarded as a valid and reliable measure of health as it encompasses the subjective experience of fatal and nonfatal diseases and the general feeling of well-being [3, 32]. The self-reported health variable is highly correlated with objective measures of health, such as mortality, morbidity, or diagnosis from a clinical exam. The self-rated health measure is a salient predictor of morbidity and mortality [33], and an even stronger predictor of physical health, mortality, and chronic diseases [14].

Education was measured by asking, ‘What is the highest degree you have completed?’ Respondents could select one of the following answer categories: ‘not received education,’ ‘primary school,’ ‘junior high school/professional high school,’ ‘senior high school,’ ‘junior college,’ ‘college,’ and ‘undergraduate.’ For individuals who were still attending school, they were asked which year they attended at the time of the survey. The answers ranged from 0 years to 22 years. Given that adults aged 24 years or younger may not have completed their educational careers by the time they were surveyed, we selected samples with respondents aged 22 years or older in an attempt to avoid assessing effects of education on health prematurely [21, 34].

The cohort variable was constructed by asking, ‘Which year was you born?’. Based on the birth year, we constructed eight birth cohorts based on historical periods of social change in China during respondents’ formative years beginning at age 10. The cohorts included the *Children of Old China* who were born before 1939, the *Children of New China* (1939–1946), the *‘Lost’ Generation* (1947–1955), the *Children of Early Cultural Revolution* (1956–1960), the *Children of Late Cultural Revolution* (1961–1966), the *Children of Economic Reform* (1967–1976), the *Children of Early Opening-Ups* (1977–1983), and *the Children of Late Opening-Ups* (1984–1994) [35].

Additionally, employment status, family income, and frequency of physical exercise were important factors affecting health [36, 37], so we used them as control variables. For employment status, we used the ‘not employed’ answer as the reference category. We used average family annual income to measure economic background. Family income variable consists of operating income, wage, transfer income, property income and other income. The frequency of physical exercise was measured by asking, ‘how often do you exercise in the last month?’ The answer options ranged from ‘never,’ ‘one time a month,’ ‘two or three times a month,’ ‘two or three times a week, and ‘almost every day,’ and they were coded as 0 to 4 respectively. Table 1 show the descriptive statistics of variables.

**Table 1:** Descriptive Statistics of Variables in the Analytic Sample^1^

	Frequency	Percent
Gender		
Female	14,017	50.8%
Male	13,563	49.2%
Self-rated health		
Good Health	22,450	81.4%
Poor Health	5130	18.6%
Education Level^2^		
Less than MS (1~11 years)	13,977	50.7%
Middle School (< 12 years)	8078	28.5%
High School (12 years)	3396	12.3%
Beyond HS (> 12 years)	2129	7.7%
Employed or Not		
Employed	11,970	43.4%
Not Employed	15,610	56.6%
Never	5212	18.9%
Frequency of physical exercise		
One time a month	303	1.1%
Two or three times a month	952	3.45
Two or three times a week	2455	8.9%
Almost everyday	18,672	67.7%
	Mean	Standard Deviation
Age	49.6	14.323
Education year	6.9	4.677
Log Family income	8.7	1.111

^1^This table reports sample statistics of 27,580 unique respondents

^2^The education level variable is for descriptive purpose only. We used the continuous variable (Education Year) in the analysis

### Models

In this study, we want to estimate how education effect change within individuals (over survey waves) and between individuals (between male and female, and across birth cohorts). We used latent growth curve model (LGM) rather than a regular hierarchical model, because a multilevel model cannot map the age sequence of self-rated health for short-term panel data. A hierarchical model treats survey waves as period effects instead of treating them as continuous age graded trajectories [38]. Since CFPS is a longitudinal data, the latent growth curve model is appropriate to study both between-person and within-person changes. In our case, LGM estimates cohort variations in health across age groups as well as within-individual age trajectories over the four survey waves [39, 40].

Like other structural equation modeling, the latent growth curve modeling has two main components – the first component is the fixed effects (i.e. the coefficients) and the second component is the random variation, which estimates the amount of health variation that is unexplained. For LGM, the fixed component is further divided into two parts. First part is the *intercepts* component which estimates the between-individual differences in health across cohorts, gender, age groups. The second part is the *slopes* or growth rate component which estimate within-individual health trajectories over time, i.e. across survey waves. In practice, LGM treats individual respondents as groups and survey waves as the age sequence vectors. LGM first conducts a series of regression for all 100,608 individuals, where each individual has a regression line based on at least 3 age sequence observations. And each individual regression line has two parameters - an intercept and a slope. Then, LGM uses those intercepts and slopes to estimate the parameters for the entire sample.

Since self-reported health is a dichotomous variable in our study, the functional form for our linear growth curve model is a logistic regression, which estimates the probability of being in good health, adjust for within-person differences at the slope level or level 1 and adjust for between-person changes at the intercept level or level 2. This model estimates the gender differences for both cohort variations and age trajectories in the association between education and health over the life course. We formulate a series of linear growth curve models using HLM 7.1 software. The full model (Model 4) that controls for all interaction terms and covariates is described as follows:

The level-1 model characterizes within-individual change across survey waves after controlling for a series of variables at level-1.

$$ \mathit{\log}\left[{\varphi}_{t,i}/\left(1-{\varphi}_{t,i}\right)\right]={\eta}_{t,i}={\pi}_{0,i}+{\pi}_{1,i}\ast \left({T}_{t,i}\right) $$

Where *φ*_*t*, *i*_ is the probability of self-rated health for individual *i* at time *t* (i.e., survey wave), *π*_0, *i*_ is the intercept component, and *π*_1, *i*_ indicates the linear growth rates or the slope component. *T*_*t*, *i*_ denotes the difference between the current survey year and the reference survey year (i.e., 2010).

The level-2 model estimates the between-individual change in health with age and assesses whether there are patterns in the association between education and health in the age trajectory for gender across different cohorts. Level-2 consists of an intercept component that measures fixed effects for all individuals and a slope equation (i.e., linear growth rate) that measures the changes in fixed effect over time.

The intercept *π*_0, *i*_ equation is expressed as the following:

$$ {\pi}_{0,i}={\beta}_{0,0}+{\beta}_{0,1} Ag{e}_i+{\beta}_{0,2} Ag e\_S{q}_i+{\beta}_{0,3} Mal{e}_i+{\beta}_{0,4} Ed{u}_i+{\beta}_{0,5}\left( Mal{e}_i\ast Ed{u}_i\right)+{\beta}_{0,j}\sum \limits_{j=6}^{12} Cohor{t}_i+{\beta}_{0,k}\sum \limits_{k=13}^{19}\left( Cohor{t}_i\ast Ed{u}_i\right)+{\beta}_{0,20} Incom{e}_i+{\beta}_{0,21} Employe{d}_i+{\beta}_{0,22} Exercis{e}_i+{\gamma}_{0i} $$

Where *β*_0, 0_ denotes the overall probability of reporting the health of all individuals across survey waves. *β*_0, 1_ to *β*_0, 19_ are the fixed coefficients, including the main effects of age, age squared, gender, education, and cohort, and the interaction effects between gender and education and between cohort and education. *β*_0, 20_ to *β*_0, 22_ denote coefficients for level-2 covariates: family income, employee status, and frequency of physical exercise. *γ*_0*i*_ denotes the variance component for the fixed intercept equation.

The linear growth rate of the period *π*_1,*i*_ equation is expressed as the following:

$$ {\pi}_{1,i}={\beta}_{1,0}+{\beta}_{1,1} Ag{e}_i+{\beta}_{1,2} Mal{e}_i+{\beta}_{1,3} Ed{u}_i+{\beta}_{1,4}\left( Mal{e}_i\ast Ed{u}_i\right)+{\beta}_{1,j}\sum \limits_{j=5}^{11} Cohor{t}_i+{\beta}_{1,k}\sum \limits_{k=12}^{18}\left( Cohor{t}_i\ast Ed{u}_i\right)+{\beta}_{1,19} Incom{e}_i+{\beta}_{1,20} Employe{d}_i+{\beta}_{1,21} Exercis{e}_i $$

The slope component includes all of the corresponding variables from the intercept component, except for *Age* _ *Sq*_*i*_ because we assume the rate of change in age effect is the same across all survey waves. We also do not control for random effect for the slope equation, because we lack the statistical power and we assume the slope coefficients are fixed for all respondents. Lastly, we examine the full model separately for each gender, allowing us to compare the significance of association and general direction between the female-only model (Model 6) and the male-only model (Model 7).

## Results

### Gender difference: results for the resource substitution hypothesis

Table 2 shows the descriptive statistics for all the variables in the analytic models. As expected, men on average report better health status, higher educational attainment, higher income, and higher physical exercise rates than their female counterparts. The sample size for each cohort by gender is shown in Table 3.

**Table 2:** Descriptive Statistics of Variables in the Analysis by Gender

	Male	Female
Sample Size	49,213	51,395
Self-rated health (good health = 1)	85.10%	77.80%
Age	47.13 (14.28)	46.61 (14.369)
Education year	7.79 (4.288)	5.99 (4.814)
Log Family income	8.67 (1.106)	8.65 (1.244)
Employed	64.40%	49.20%
Frequency of physical exercise (0–4)	3.10 (1.523)	3.00 (1.607)

Note: Standard Deviation in parentheses

**Table 3:** Sample Size for Each Cohort by Gender (Observations)

	Full sample	Male	Female
Sample Size	100,608	49,213	51,395
Old China: 1908–1938	4476 (4.4%)	2122 (4.3%)	2354 (4.6%)
New China: 1939–1946	8193 (8.1%)	4308 (8.8%)	3885 (7.6%)
The ‘Lost’ Generation: 1947–1955	18,165 (18.1%)	8886 (18.1%)	9279 (18.1%)
Early Cultural Revolution: 1956–1960	9693 (9.6%)	4868 (9.9%)	4825 (9.4%)
Late Cultural Revolution: 1961–1966	15,110 (15%)	7205 (14.6%)	7905 (15.4%)
Economic Reform: 1967–1976	23,385 (23.2%)	11,224 (22.8%)	12,161 (23.7%)
Early Opening-Ups: 1977–1983	11,020 (11.0%)	5441 (11.1%)	5579 (10.9%)
Late Opening-Ups: 1984–1994	10,566 (10.5%)	5159 (10.5%)	5407 (10.5%)

Note: Percentage in parentheses

In Table 4, we present the results from a series of age vector models. Based on the odds ratio in Model 2 for males, men reported better health than women. This model tests the resource substitution hypothesis, which supposes that there is a substantial educational difference in the slope of gender (Model 1). For male respondents, a one-year increase in education yields a change in log odds of 0.07 or an odds ratio of 1.073. Thus, a female with one additional year of education is on average 1.012 times more likely than a male with one additional year of education to report a status of healthy in the survey holding all else constant. In other words, the association between education and health is weaker among men than among women.

**Table 4:** Coefficients and Odds Ratios of Linear Growth Curve Models for Education, Gender and Cohort Effects on Self-Rated Health

	Full Sample															Female Only			Male Only		
	Model 1			Model 2			Model 3			Model 4			Model 5			Model 6			Model 7		
	Coef.	O.R.	P	Coef.	O.R.	P	Coef.	O.R.	P	Coef.	O.R.	P	Coef.	O.R.	P	Coef.	O.R.	P	Coef.	O.R.	P
Fixed Effect Component																					
*For Intercept*																					
Intercept Mean	0.828	2.289	***	0.697	2.008	***	1.024	2.784	***	1.162	3.195	***	−0.533	0.587	*	−0.183	0.833		−0.673	0.510	+
Age (Centered at 22)	− 0.043	0.958	***	−0.041	0.959	***	−0.047	0.954	***	−0.046	0.955	***	− 0.040	0.961	***	− 0.053	0.948	***	−0.020	0.980	*
Age Squared	0.001	1.001	***	0.001	1.001	***	0.001	1.001	***	0.001	1.001	***	0.001	1.001	***	0.001	1.001	***	0.001	1.001	**
Male	0.399	1.490	***	0.446	1.561	***	0.392	1.480	***	0.392	1.479	***	0.384	1.469	***						
Education (Years)	0.091	1.095	***	0.086	1.089	***	0.085	1.088	***	0.055	1.056	***	0.038	1.039	**	0.016	1.017		0.048	1.049	**
Male*Education				−0.016	0.984	***	−0.006	0.994		−0.004	0.996		−0.005	0.995							
*Cohort (Old China: 1908–38)*																					
New China:1939–46							−0.109	0.897		−0.171	0.843		−0.131	0.877		−0.147	0.863		−0.145	0.865	
Lost Generation: 1947–55							−0.172	0.842		−0.241	0.786	+	−0.184	0.832		−0.364	0.695	+	0.063	1.065	
Early Cultural Revolution: 1956–60							−0.318	0.728	+	−0.504	0.604	**	−0.435	0.648	*	−0.724	0.485	**	0.035	1.035	
Late Cultural Revolution: 1961–66							−0.416	0.659	*	−0.476	0.621	*	−0.357	0.700	+	−0.739	0.478	**	0.290	1.336	
Economic Reform: 1967–76							−0.420	0.657	+	−0.709	0.492	**	−0.510	0.601	*	−0.989	0.372	**	0.242	1.274	
Early Opening-Ups: 1977–83							−0.366	0.693		−0.702	0.496	*	−0.388	0.678		−1.021	0.360	**	0.546	1.726	
Late Opening-Ups: 1984–94							−0.131	0.877		−0.127	0.881		0.246	1.279		−0.279	0.756		0.804	2.234	
Interaction: Education*Cohort																					
Education*New China										0.016	1.016		0.021	1.022		0.038	1.038		0.025	1.025	
Education*Lost Generation										0.013	1.013		0.017	1.017		0.040	1.040		0.004	1.004	
Education*Early Cultural Revolution										0.038	1.038	*	0.045	1.046	**	0.061	1.063	*	0.026	1.027	
Education*Late Cultural Revolution										0.018	1.018		0.022	1.023		0.038	1.039		0.002	1.002	
Education*Economic Reform										0.057	1.058	***	0.059	1.061	***	0.084	1.088	***	0.033	1.033	
Education*Early Opening-Ups										0.062	1.063	***	0.058	1.059	**	0.097	1.102	***	0.022	1.022	
Education*Late Opening-Ups										0.024	1.024		0.019	1.020		0.039	1.040		0.027	1.027	
Family Income													0.157	1.170	***	0.158	1.172	***	0.155	1.168	***
Employed													0.262	1.299	***	0.166	1.180	***	0.393	1.481	***
Physical Exercise													0.050	1.051	***	0.051	1.052	***	0.049	1.051	**
*For Linear Growth Rates or Slope (Timestamp = Survey Waves 2010–2016)*																					
Slope Mean	− 0.009	2.289		−0.016	0.984	***	−0.059	0.943	+	−0.057	0.945		0.048	1.049		0.016	1.016		0.113	1.119	+
Age (Centered at 22)	−0.001	0.999					0.001	1.001		0.001	1.001		0.000	1.000		0.002	1.002		−0.002	0.998	
Male	−0.002	0.998					0.016	1.016		0.016	1.016		0.017	1.017							
Education (Years)	−0.001	0.999					0.001	1.001		0.001	1.001		0.002	1.002		0.005	1.005		−0.003	0.997	
Male*Education							−0.003	0.997	*	−0.003	0.997	*	−0.003	0.997	*						
*Cohort (Old China: 1908–38)*																					
New China:1939–46							0.004	1.004		0.014	1.014		0.005	1.005		0.012	1.012		−0.002	0.998	
Lost Generation: 1947–55							0.017	1.017		0.004	1.004		−0.014	0.986		0.011	1.011		−0.048	0.953	
Early Cultural Revolution: 1956–60							0.035	1.036		0.044	1.045		0.020	1.020		0.072	1.075		−0.077	0.926	
Late Cultural Revolution: 1961–66							0.051	1.052		0.031	1.032	+	−0.002	0.998		0.036	1.037		−0.048	0.953	
Economic Reform: 1967–76							0.076	1.079	+	0.084	1.087		0.040	1.041		0.099	1.104		−0.048	0.953	
Early Opening-Ups: 1977–83							0.085	1.089		0.093	1.098		0.034	1.034		0.092	1.096		−0.060	0.942	
Late Opening-Ups: 1984–94							0.022	1.023		0.048	1.050		−0.019	0.981		−0.024	0.976		0.054	1.055	
Interaction: Education*Cohort																					
Education*New China										−0.003	0.997		−0.003	0.997		−0.005	0.995		−0.003	0.997	
Education*Lost Generation										0.003	1.003		0.003	1.003		0.000	1.000		0.003	1.003	
Education*Early Cultural Revolution										−0.002	0.998		−0.003	0.997		−0.006	0.994		0.002	1.002	
Education*Late Cultural Revolution										0.003	1.003		0.002	1.002		−0.001	0.999		0.001	1.001	
Education*Economic Reform										−0.002	0.998		−0.002	0.998		−0.007	0.993		0.000	1.000	
Education*Early Opening-Ups										−0.002	0.998		− 0.002	0.998		−0.001	0.999		−0.004	0.996	
Education*Late Opening-Ups										−0.003	0.997		−0.004	0.996		0.000	1.000		−0.018	0.982	*
Family Income													−0.006	0.994		−0.007	0.993	+	−0.004	0.996	
Employed													−0.009	0.991	*	− 0.006	0.994		−0.014	0.986	
Physical Exercise													−0.008	0.992		− 0.008	0.992	**	−0.007	0.993	*
Total Variance	1.617			1.677***			1.679***			1.679***			1.658***			1.605***			1.723***		
Sample Size	100,608															51,395			49,213		

### Cohort variations in education and health: results for the rising importance hypothesis

In order to understand the cohort variations in self-reported health, we added the cohort variables in Model 3. The odds ratios for each cohort in Model 3 shows that the younger cohort reports healthier than the oldest cohort, but only the health for the Late Cultural Revolution (1961–1966) cohort is statistically significant from that of the oldest cohort.

To test the rising importance hypothesis, which supposes that the association between education and health becomes stronger over time for a given population, we included interaction effects between education and cohort in Model 4. We found that the association between education and health did not change significantly for cohorts before 1955, and the association became stronger for younger cohorts. For the 1956–1960 cohort, a one-unit increase in education increased the odds of reporting good health by 3.8% (*P* < 0.05). The odds ratio for the 1908–1938 cohort was 1.058 (*P* < 0.001). The odds ratio of a one-unit education gain for cohort 1956–1960 over the odds ratio of a one-unit education gain for the 1908–1938 cohort was 1.063 (*P* < 0.001). In other words, respondents from cohort 1956–1960 with one additional year of education were on average 1.038 times more likely than respondents from cohort 1908–1938 with one additional year of education to report healthy in the survey holding all else constant. With one additional year of education, respondents from the 1967–1976 cohort and the 1977–1983 cohort were on average 1.058 times and 1.063 times respectively more likely to report as healthy compared with respondents from the 1908–1938 cohort. We also noted that the rising trend disappeared in the youngest cohort.

### Gender difference in education and health across cohorts

We also established full models separately for men and women. Model 4 showed that the odds ratios of the interaction between education and cohort were significantly more than 1 among women for the 1956–1960, 1967–1976 and 1977–1983 cohorts. The results in Model 5 suggested that education’s positive effects on health increased for the 1956–1960, 1967–1976 and 1977–1983 cohorts. Female respondents from the 1956–1960, 1967–1976 and 1977–1983 cohorts with one additional year of education were on average 1.063 times, 1.088 times, and 1.102 times more likely than respondents from the 1908–1938 cohort (*P* < 0.05) to report better health, respectively. The pattern for the relationship between education and health for the female subsample was the same as that of the full sample. As with the rising importance hypothesis, the rising trend also disappeared in the youngest cohort. In Model 6, education was not positively related to good health for men (*P* > 0.1). The interactional effects between education and cohort were not statistically significant. In other words, the education effects were the same across all eight cohorts among men, but there was a gender difference in education and health across cohorts. The gender difference became statistically significant in the slope model, but it was not statistically significant in the intercept model.

## Discussion

This study tests the gender disparity in the education-health relationship across cohorts in China and assesses the resource substitution hypothesis and rising importance hypothesis in the Chinese context. Results reveal that the education effect on health is stronger among women than among men, which means the resource substitution hypothesis is supported in the Chinese context, and the result is consistent with related U.S. studies. However, compared to previous findings about the rising importance hypothesis from the United States, there are two notable differences in the Chinese context. First, the effect of education on health has not increased from the oldest cohort to the youngest cohort, and the gaps in health remained stable for some cohorts. Second, for the rising importance hypothesis, there is a gender difference in the educational effects on health across cohorts in the Chinese context.

We think potential explanations for the U.S.-China differences lie in the role of sociocultural and policy change. There are two crucial reasons which can explain the rising importance phenomenon in U.S. First is the relationship between education and income has intensified over the years [23], and the same intensity also occurred in the relationship between the educational and health-related behaviors [8]. Another reason is the education expansion resulting in an increased adverse selection of the lower-educated individuals, which resulted in the expanding inequality in health-related resources between highly educated and uneducated individuals [41]. However, in China the association between education and health-related resources may be one of the reasons for the complex trend across cohorts. Eating dinner and drinking wine with friends, colleagues, and superiors is a crucial way to maintain a social network and to obtain resources in Chinese society but can be a negative effect for health outcomes [42].

Before the Reform and Opening-Up, individuals had limited access to food and clothing under the socialist economy. After decades of Opening-Up, more people began to enjoy abundant material prosperity [43]. Individuals with higher education and more purchasing power were more likely to participate in social gatherings, indulge in unhealthy diets, and consume more alcohol [40]. Furthermore, these behaviors are empirically found to be more pronounced among men than women [42], which could possibly be supported by a strong positive relationship between education and drinking in the male sample in our study.

As for the cohort patterns, we argued that the drastic educational landscape changes in China affect the relationship between education and health-related resources. The rapid increase in social and economic benefits from education for the 1967–1976 cohort may be due to the recovery of the college entrance exam in 1977. Although there were less than 1.4 million students who graduated from college prior to 2003, credentials became more critical for finding jobs after the Opening-Up and Reform periods in 1978. Members of the 1967–1976 and 1977–1983 cohorts who graduated from college could acquire well-paying jobs compared with cohort members who did not receive a college degree. However, with educational expansion starting in 1999 in China, the number of people receiving a college education had been increasing rapidly. According to the Chinese Educational Statistics Report (2018), the number of people receiving a college education rose from 1.08 million in 1998 to 7.62 million in 2017 [28]. Compared with older cohorts, the children of Later Opening-Ups (1984–1994) could receive an education more easily, but the valuation of the same academic degree had decreased. Educational expansion resulted in the phenomenon of over-education, which lessens the income benefits from educational attainment due to the mass of individuals who have similar credentials vying for similar, limited opportunities. Members of the youngest cohort faced more competition in the labor market than the members of the Early Opening-Ups, for whom the adverse effects from educational expansion had not caught up. Educational expansion weakens the role of education in improving access to both socioeconomic and health-related resources [30, 44]. The rate of returns for education even declined in 2009 [25]. As a result, the rising trend disappeared for the 1984–1994 cohort.

The gender difference in education and health across cohorts can be attributed to women experiencing greater difficulty with gaining access to educational and health-related resources. Results show that the links between education and health are stronger among women than among men in China, which support the ‘resource substitution hypothesis’ from the perspective of cohorts. Women have been in a socially disadvantaged position for decades [45], so they have fewer resources to rely on. On the contrary, men have more resources presently and historically, so education is less important for men than for women. Another possible reason for this gender disparity is females’ disadvantages in the labor market. Due to governmental deregulation of the market, enterprises begin to employ more men than women, and discrimination against women in the labor market has increased since the “reform and open-up” [46, 47]. Moreover, education is more important for women in securing jobs than for men. Hence, education is more important for women than for men across cohorts.

The strength in the research is revealing the gender difference in education-health pattern, investigating the gender difference in the education-health patterns across cohorts, especially for the recent cohorts, and finding the difference between U.S. and China. However, there are also limitations in the research. The observation period has only 6 years. Thus, the age overlaps between cohorts – the points at which actual cohort effects can be identified – are small. Hence, an age vector graph can be useful to identify any age trajectories difference in cohort effects.

Although self-reported health is a valid measure of health status [32], it is susceptible to effects based on individual characteristics and cultural contexts. A study found that people with higher education and income tend to report their health optimistically in China [48]. Given its vulnerability to individual and social influences, the association between education and self-reported health may be receiving too much attention in the literature. The vignette method could be incorporated into studies in the future to provide more qualitative information if related items are included in the design of data collection instruments. The relationship between education and health is complex or potentially reciprocal - people with higher education can improve their health, but people may drop out of high school due to severe health problems. The primary goal of our study is to illustrate the demographic and cohort patterns for the education and health association. As such, we cannot establish a causal relationship between education and health, however different methods and data may allow for the conclusion of causal inferences in future studies.

## Conclusions

In conclusion, the results show that the education-health relationship is stronger among women than among men, and there is gender difference in the education-health patterns across cohorts. This different pattern suggests that broad contextual factors such as gender and cohort can significantly shape the education-health patterns in China. Our findings show that the gender difference in the association between education and health is significant, but China’s unique history of educational and health development and cohort-specific formulative experience, may have also influenced these education-health patterns. Future studies may consider different theoretical frameworks, such as social transformation theory, to explain the gender disparity in educational benefits across cohorts.

## Data Availability

The datasets generated and /or analyzed during the current study are available in the Chinese Family Panel Survey, http://www.isss.pku.edu.cn/cfps/index.htm.

## Abbreviations

CFPS: Chinese Family Panel Survey
LGM: Linear Growth Curve Modeling
